# Anti-inflammatory agents and substance P depletion in experimental ileitis

**DOI:** 10.1155/S0962935193000407

**Published:** 1993

**Authors:** M. J. S. Miller, H. Sadowska-Krowicka, S. Chotinaruemol, M. Wong, D. A. Clark, A. Y. Jeng

**Affiliations:** 1 Department of Pediatrics Louisiana State University Medical Center New Orleans LA 70112 USA; 2 Research Department Ciba-Geigy Pharmaceuticals Summit NJ 07901 USA

## Abstract

To understand the interactions between substance **P** and gut inflammation, changes in substance **P** levels were evaluated in a chronic model of ileitis in response to three anti-inflammatory agents with distinct mechanisms of action. The agents were the prostaglandin E_1_ analogue misoprostol (30 μg/kg, s.c., b.i.d.), the nitric oxide synthase inhibitor N^G^-nitro-L-arginine methyl ester (L-NAME, 100 μg/ml in drinking water) and the leumedin, N-(fluorenyl-9-methoxycarbonyl)-L-leucine (NPC 15199, 10 mg/kg, s.c.). Ileitis was induced by a transmural injection of trinitrobenzene sulphonic acid (TNBS 30 mg/kg in 50% ethanol) into the distal ileum of guinea-pigs. All anti-inflammatory therapies were introduced after TNBS administration and continued until day 7, when guinea-pigs were killed. Ileal substance **P** levels were measured by radioimmunoassay, and granulocyte infiltration was quantified by myeloperoxidase (MPO) activity. Protein and nitrite (an index of nitric oxide formation) levels in a luminal saline lavage were quantified in all groups. TNBS ileitis caused a marked reduction in ileal substance **P** content and increased MPO activity, protein and nitrite secretion. The nitric oxide synthase inhibitor, L-NAME, completely restored all parameters to baseline. Misoprostol attenuated the granulocyte infiltration and exacerbated protein leak but had no effect on substance **P** levels. In contrast, NPC 15199 had no effect on granulocyte infiltration but normalized substance **P** levels and protein leak. Only L-NAME and NPC 15199 blocked the TNBS induced increase in nitrite levels. These results suggest that the regulation of granulocyte infiltration in this model is unrelated to changes in substance **P** levels. Inhibition of nitric oxide synthase was the most effective therapeutic strategy in TNBS ileitis but the precise interactions between nitric oxide and the enteric nervous system during inflammatory states remain to be defined.


Mediators of Inflammation 2, 293–297 (1993)

### Anti-inflammatory agents and substance P depletion in experimental ileitis

M. J. S. Miller,[1,CA] H. Sadowska-Krowicka,[1] S. Chotinaruemol,[1] M. Wong,[2] D. A. Clark[1] and A. Y. Jeng[2]

[1] Department of Pediatrics, Louisiana State University Medical Center, New Orleans, LA 70112, USA; [2] Research Department, Ciba-Geigy Pharmaceuticals, Summit, NJ 07901, USA

[CA] Corresponding Author

**To understand the interactions between substance P and gut inflammation, changes in substance P levels were evaluated in a chronic model of ileitis in response to three anti-inflammatory agents with distinct mechanisms of action. The agents were the prostaglandin $E_1$ analogue misoprostol (30 μg/kg, s.c., b.i.d.), the nitric oxide synthase inhibitor $N^G$-nitro-L-arginine methyl ester (L-NAME, 100 μg/ml in drinking water) and the leumedin, *N*-(fluorenyl-9-methoxycarbonyl)-L-leucine (NPC 15199, 10 mg/kg, s.c.). Ileitis was induced by a transmural injection of trinitrobenzene sulphonic acid (TNBS 30 mg/kg in 50% ethanol) into the distal ileum of guinea-pigs. All anti-inflammatory therapies were introduced after TNBS administration and continued until day 7, when guinea-pigs were killed. Ileal substance P levels were measured by radioimmunoassay, and granulocyte infiltration was quantified by myeloperoxidase (MPO) activity. Protein and nitrite (an index of nitric oxide formation) levels in a luminal saline lavage were quantified in all groups. TNBS ileitis caused a marked reduction in ileal substance P content and increased MPO activity, protein and nitrite secretion. The nitric oxide synthase inhibitor, L-NAME, completely restored all parameters to baseline. Misoprostol attenuated the granulocyte infiltration and exacerbated protein leak but had no effect on substance P levels. In contrast, NPC 15199 had no effect on granulocyte infiltration but normalized substance P levels and protein leak. Only L-NAME and NPC 15199 blocked the TNBS induced increase in nitrite levels. These results suggest that the regulation of granulocyte infiltration in this model is unrelated to changes in substance P levels. Inhibition of nitric oxide synthase was the most effective therapeutic strategy in TNBS ileitis but the precise interactions between nitric oxide and the enteric nervous system during inflammatory states remain to be defined.**

**Key words:** Granulocytes, Ileitis, Inflammation, Nitric oxide, Substance P

### Introduction

A model of chemical ileitis, induced by intraluminal trinitrobenzene sulphonic acid (TNBS) in a 50% ethanol solution has been developed.[1,2] This model is an adaptation of the TNBS colitis model used by several groups.[3,4] TNBS ileitis is associated with a marked granulocyte and mononuclear infiltration, submucosal fibrosis, crypt, smooth muscle and mast cell hyperplasia, protein and fluid secretion. These responses peak around 7 days post-TNBS, with a gradual restoration of structure and function within 1 month.[1,2] Although clearly an artificial means of inducing subchronic inflammation, this model does bear a resemblance to Crohn's disease.

Because inflammation is induced in the guinea-pig ileum, there is the opportunity to evaluate interactions between substance P and the inflammatory processes, as substance P innervation has been extensively characterized in the guinea-pig ileum.[5] It has recently been shown that in TNBS ileitis there is a sustained loss of intestinal substance P content which parallels the time-course of the other indices of ileitis. This reduction in substance P was confined to the mucosa/submucosa and in particular, perivascular innervation.[6] Peptide neurotransmitters including CGRP, VIP, and substance P, have been evaluated in models of gut inflammation and clinical specimens, but a consensus as to how these neurotransmitters participate in or respond to the inflammatory process, has not been reached.[7–9]

In order to examine how the changes in substance P levels in ileitis, the effects of three anti-inflammatory agents which differ markedly in

© 1993 Rapid Communications of Oxford Ltd structure and mechanism of action were compared. The anti-inflammatory agents chosen were misoprostol, a $PGE_1$ analogue with various cytoprotective properties;[10,11] the leumedin, NPC 15199, which has diverse anti-inflammatory and immunosuppressive actions;[12,13] and L-NAME, a nonspecific inhibitor of nitric oxide synthase.[14] Under acute circumstances, inhibition of nitric oxide formation exacerbates granulocyte infiltration and inflammation.[15] However, it has been noted that in the TNBS model of ileitis, nitric oxide synthase inhibition ameliorated the inflammation.[1] Thus, nitric oxide synthase inhibitors can display both pro- and anti-inflammatory properties, depending on the duration of administration and pathophysiology and possibly the enzyme source of nitric oxide.[1,15] The aim of the study was to identify components of the inflammatory process which interact with the enteric nervous system and influence substance P dependent mechanisms.

### Materials and Methods

*TNBS ileitis:* The characteristics of this model in guinea-pigs have recently been described.[1,2,6] Under aseptic surgical conditions fasted female Hartley guinea-pigs (250–350 g) were anaesthetized with ketamine (40 mg/kg) and the distal ileum isolated after a midline laparotomy. TNBS (30 mg/kg in 50% ethanol) was injected into the lumen of ileum by transmural injection using a 26 gauge needle. Control animals received an equivalent volume of saline (sham) or 50% ethanol (vehicle). The abdominal incision was sutured and the guinea-pig was transferred to a heated waterbed for postoperative recovery. Guinea-pigs were anaesthetized 7 days later as described above. The distal ileum was ligated into a loop, commencing 15 cm from the ileocaecal valve, 2 ml of saline was injected intraluminally and after 30 min the loop fluid was removed. Loop fluid volume was recorded, the fluid was centrifuged to remove particulate matter, and the supernatant divided into aliquots and frozen. Sections of ileum were used for estimation of MPO activity and SP content.

Misoprostol was administered subcutaneously, twice daily (30 mg/kg, s.c., b.i.d.) for 7 days, the first dose was given 20 min before ileitis induction. L-NAME was administered in the drinking water (100 μg/ml *ad libitum*) and NPC 15199 was administered by subcutaneous injection (10 mg/kg) daily for 7 days.

*Substance P content:* Approximately 1 g of tissue from each loop was separated, chopped, weighed and then boiled in 5 ml/g of tissue wet weight of 0.1 N HCl for 10 min. The tissue was then sonicated and centrifuged at 1 300 × *g* for 10 min. The supernatant was collected, coded and frozen at −70°C until levels of substance P were measured by radioimmunoassay (RIA). The pH of supernatants was normalized (pH 7.0) prior to RIA, which was conducted without knowledge of the experimental protocol.

Substance P and substance P antiserum were obtained from Cambridge Research Biochemicals (Valley Stream, NY). $^{125}$I-labelled SP was purchased from New England Nuclear (Boston, MA). Goat anti-rabbit gamma-globulin antibodies and normal rabbit serum were from Calbiochem (LaJolla, CA). The substance P RIA was carried out at room temperature as described previously.[16] Briefly, samples were diluted in 300 μl of RIA buffer (0.2% BSA, 0.1% Triton X-100, and 0.02% $NaN_3$ in PBS) and mixed with 100 μl each of 1:200 diluted substance P antiserum and $^{125}$I-labelled substance P (10 000 cpm/tube). After 2 h incubation, 100 μl each of the goat anti-rabbit gamma-globulin antibodies and the normal rabbit serum were added and incubated further for 45 min. The antigen–antibody complex was then precipitated with 1 ml of 17.5% polyethylene glycol and centrifuged in a tabletop centrifuge. The supernatant was decanted and the radioactivity in the pellet was counted in a gamma counter. The total binding and nonspecific binding were measured in the absence of nonradioactive substance P and substance P antiserum, respectively. In general, the radioactivities measured in the total and nonspecific binding were 6 000 and 300 cpm, respectively.

*Myeloperoxidase activity:* Intestinal tissue (100–250 mg) was finely minced at 4°C and homogenized with a Brinkman polytron for 20 s in 50 mM hexadecyltrimethylammonium bromide to negate the pseudoperoxidase activity of haemoglobin. The homogenate was centrifuged at 20 000 × *g* for 15 min. The pellet was frozen and thawed. This cycle of homogenization and freeze/thawing was repeated twice. An extract of the final supernatant (100 μl) was mixed with 2.9 ml of potassium phosphate buffer (50 mM, pH 6.0) containing 0.167 mg/ml of O-dianisidine dihydrochloride and 0.0005% of hydrogen peroxide. Absorbance at 460 nm was determined on a Beckman DU-64 spectrophotometer over 3 min. One unit of myeloperoxidase activity was defined as that required to degrade 1 μmol of $H_2O_2$ per minute at 25°C. This method has been previously described for this model.[1,2,6]

*Ileal lavage analysis:* Fluid removed from 30 min saline lavages was analysed for protein content with a spectrophotometric method as described previously.[1,2,6] Loop fluid nitrite levels were used as an index of nitric oxide production. Nitrite levels were quantified with the Griess reaction as described previously.[1,2,6] Both lavage protein and nitrite levels were normalized for loop weight.

### Results

*Myeloperoxidase activity:* One week after TNBS administration ileal MPO activity was markedly elevated above sham-operated controls (Fig. 1). Concomitant treatment with misoprostol attenuated granulocyte infiltration whereas L-NAME abolished the response, i.e. MPO levels were equivalent to the basal values found in sham-operated controls. In contrast, NPC 15199 did not affect TNBS induced increases in ileal MPO activity (Fig. 1). The authors noted that higher concentrations of NPC 15199 (100 mg/kg s.c.) prevented the increased MPO activity following TNBS. However, these results were not detailed in this study. Thus, the observations with NPC 15199 are consistent with published reports on its ability to inhibit neutrophil–endothelium interactions.[12,13] The high dose of NPC 15199 also restored substance P levels (data not shown).

*Substance P content:* Whole intestinal substance P content is reduced by approximately 40% at day 7 of TNBS treated animals (Fig. 2). Misoprostol administration had no effect on TNBS induced reductions in substance P content. However, both L-NAME and NPC 15199 restored substance P levels to those found in sham controls (Fig. 2).

*Ileal lavage protein and nitrite levels:* TNBS ileitis was associated with a marked increase in lavage protein levels (Fig. 3). This response was exacerbated by misoprostol ($p < 0.05$) but completely abolished by either L-NAME or NPC 15199 ($p < 0.02$).

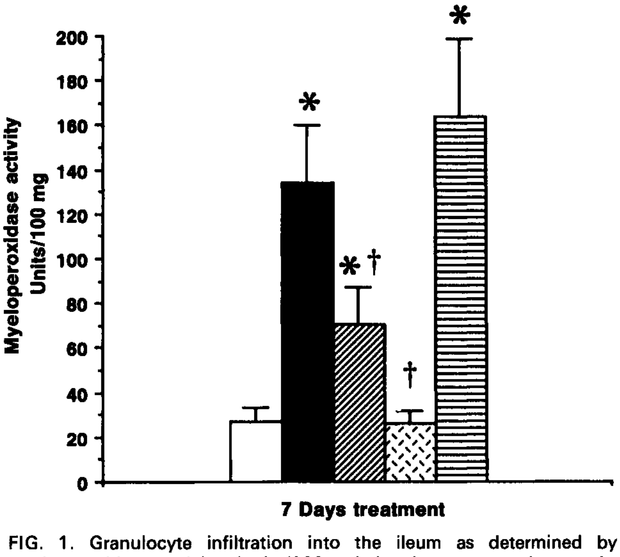

FIG. 1. Granulocyte infiltration into the ileum as determined by myeloperoxidase activity (units/100 mg) in sham operated controls, TNBS induced ileitis and TNBS with concomitant therapy with misoprostol (30 mg/kg, s.c., b.i.d.), L-NAME (100 μg/ml in drinking water, *ad libitum*) or NPC 15199 (10 mg/kg, s.c.). All groups were treated for 7 days. * Significant difference from control (sham) animals ($p < 0.05$). † Significant difference between TNBS alone *vs.* TNBS with drug therapy ($p < 0.05$). □, Control; ■, TNBS; ▨, TNBS + MISO; ▩, TNBS + L-NAME; ▤, TNBS+NPC 15199.

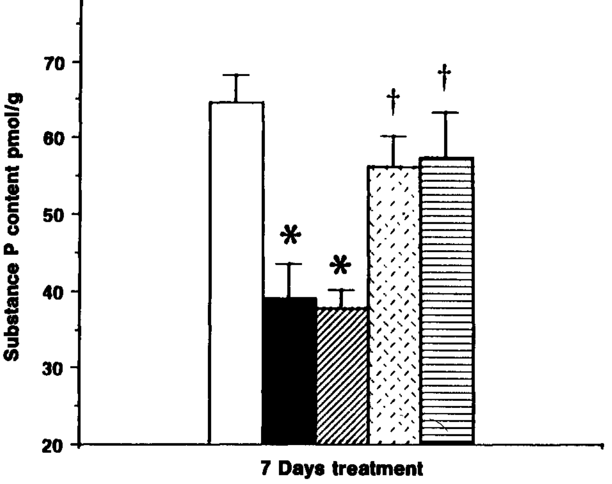

FIG. 2. Substance P content (pmol/g) in whole ileal segments determined by radioimmunoassay, in sham operated controls, TNBS induced ileitis and TNBS with concomitant therapy with misoprostol (30 mg/kg, s.c., b.i.d.), L-NAME (100 μg/ml in drinking water, *ad libitum*) or NPC 15199 (10 mg/kg, s.c.). All groups were treated for 7 days. Significant difference from control (sham) animals ($p < 0.05$). † Significant difference between TNBS treatment alone *vs.* TNBS with drug therapy ($p < 0.05$). □, Control; ■, TNBS; ▨, TNBS + MISO; ▩, TNBS + L-NAME; ▤, TNBS+NPC 15199.

Lavage nitrite levels were also elevated above baseline in TNBS ileitis ($p < 0.05$). This response was unaltered by misoprostol treatment but completely prevented by either L-NAME or NPC 15199 administration ($p < 0.05$, Fig. 4). Table 1 summarizes the markers of gut inflammation and the influence of the therapeutic approaches.

Morphologically, only L-NAME restored normal gut appearance; in either NPC 15199 or misoprostol treated animals the crypt hypertrophy, submucosal fibrosis and smooth muscle hyperplasia that are associated with TNBS ileitis were readily apparent.

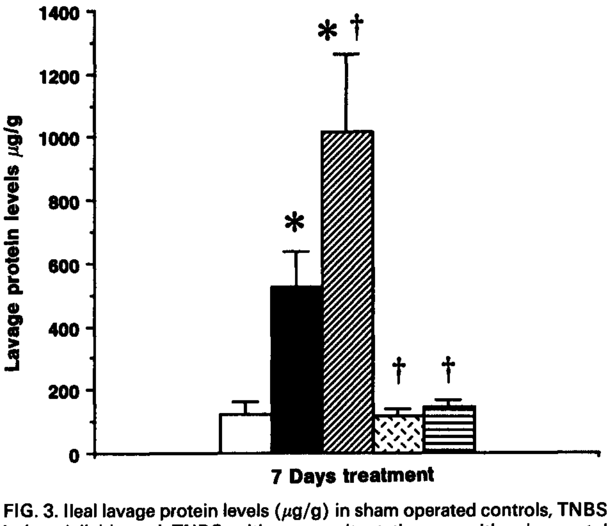

FIG. 3. Ileal lavage protein levels (μg/g) in sham operated controls, TNBS induced ileitis and TNBS with concomitant therapy with misoprostol (30 mg/kg, s.c., b.i.d.), L-NAME (100 μg/ml in drinking water, *ad libitum*) or NPC 15199 910 mg/kg, s.c.). All groups were treated for 7 days. * Significant difference from control (sham) animals ($p < 0.05$). † Significant difference between TNBS alone *vs.* TNBS with drug therapy ($p < 0.05$). □, Control; ■, TNBS; ▨, TNBS + MISO; ▩, TNBS + L-NAME; ▤, TNBS+NPC 15199.

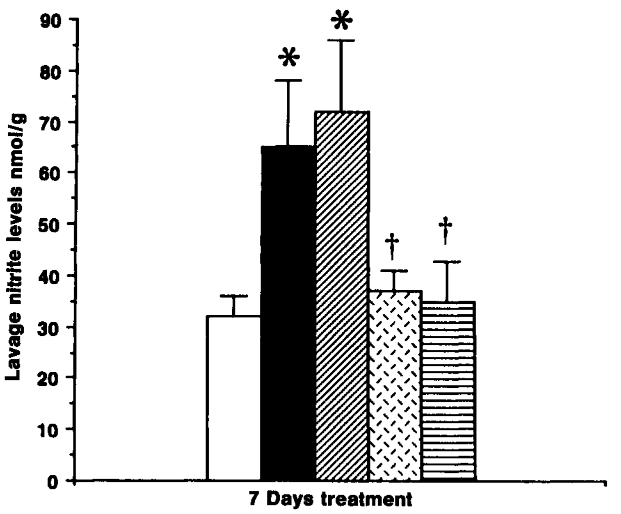

FIG. 4. Ileal lavage nitrite levels (ng/g) in sham operated controls, TNBS induced ileitis and TNBS with concomitant therapy with misoprostol (30 mg/kg, s.c., b.i.d.), L-NAME (100 μg/ml in drinking water, *ad libitum*) or NPC 15199 (10 mg/kg, s.c.). All groups were treated for 7 days. * Significant difference from control (sham) animals ($p < 0.05$). † Significant difference between TNBS alone *vs.* TNBS with drug therapy ($p < 0.05$). □, Control; ■, TNBS; ▨, TNBS + MISO; ⊠, TNBS + L-NAME; ▤, TNBS+NPC 15199.

**Table 1.** Indices of ileitis and the influence of anti-inflammatory agents

| Treatment group | Change from control animals | | | |
|---|---|---|---|---|
| | MPO | SP | Lavage protein | Lavage nitrite |
| TNBS | ↑↑ | ↓ | ↑ | ↑ |
| TNBS and misoprostol | ↑ | ↓ | ↑↑ | ↑ |
| TNBS and L-NAME | ↔ | ↔ | ↔ | ↔ |
| TNBS and NPC 15199 | ↑↑ | ↔ | ↔ | ↔ |

Arrows indicate the direction of change, the number of arrows indicate the magnitude of an alteration from control guinea-pigs.

### Discussion

One week after induction of ileitis with TNBS intestinal substance P content was markedly reduced. This primarily reflects alterations in submucosal substance P and to a lesser extent mucosal sites, because it has been noted previously that myenteric plexus substance P content remains unaltered.[6] The present study was designed to determine the relationship between these changes in ileal substance P and the inflammatory process, through the use of three structurally and mechanistically different non-steroidal anti-inflammatory agents.

Perhaps the most intriguing finding was that the degree of granulocyte infiltration was unrelated to the reductions in substance P content. This finding was revealed by comparing the effects of misoprostol and NPC 15199. Misoprostol attenuated ileal MPO levels but had no effect on the substance P response, whereas NPC 15199 had no effect on MPO levels but normalized ileal substance P levels. The nitric oxide synthase inhibitor, L-NAME, corrected both MPO and substance P, which is an important observation but does not reveal a specific relationship between granulocyte infiltration and substance P.

From these observations, it is concluded that granulocytes are unlikely to mediate the changes in substance P content. This assumes that changes in MPO activity reflect granulocyte infiltration in the gut. This assumption is generally accepted although other cell types, such as macrophages, do contain myeloperoxidase. This conclusion does not preclude other interactions between sensory neuropeptides and granulocytes. For example granulocyte derived oxidants can degrade substance P[17] and inhibit its degradation by neutral endopeptidase 24.11.[18] In addition, substance P can promote granulocyte adherence to vascular endothelium.[19] However, changes in substance P content, which primarily reflects the balance of neuronal substance P synthesis and release, appear to be regulated by factors other than granulocytes.

The nature of these factors remains unknown. However, one possibility is nitric oxide. The nitric oxide synthase inhibitor, L-NAME, restored substance P levels and all other indices of gut injury.[1] Luminal nitrite levels, which is an *in vivo* index of nitric oxide production, were also normalized by NPC 15199. This experimental leumedin was as effective as L-NAME in restoring substance P content. It is possible that their effects on substance P were mediated by a common mechanism, possibly inhibition of nitric oxide synthase. However, NPC 15199 appears to have numerous immunosuppressive and anti-inflammatory properties which could be important, particularly as intestinal substance P is known to be linked to the gut associated lymphoid tissue.[20] Nevertheless if nitric oxide is an important regulator of neuronal substance P, its source is unlikely to be granulocyte derived.[21] Nitric oxide has numerous anti-inflammatory properties, such as inhibition of granulocyte–endothelial interactions[15] and improved epithelial barrier function,[2,22] which are readily demonstrable under acute conditions. However, in chronic inflammatory states, it appears that nitric oxide is pro-inflammatory, probably via free radical mechanisms and the induction of the inducible form of nitric oxide synthase. Whether neuronal (NOS III) or immune/inducible (NOS II) nitric oxide synthase is the source of nitric oxide for these effects is unknown. Immunohistochemical localization of submucosal substance P is anatomically similar to sites of constitutive NOS activity (NOS I and III),[23] particularly perivascular innervation in guinea-pig intestine. On the other hand TNBS ileitis is associated with induction of the NOS II form of nitric oxide synthase,[24] a form of nitric oxide synthase is usually quiescent in the gut.[25] The injurious effects of nitric oxide are usually linked to the inducible form because it has the capacity to produce large amounts of nitric oxide, approximately three orders of magnitude greater than the constitutive forms of nitric oxide synthase.[13] Large amounts of nitric oxide, acting alone as a free radical, or in concert with other free radical species implicated in gut inflammation,[26] appears to be important in the pathogenesis of this model of inflammatory bowel disease, as L-NAME was clearly the superior therapeutic agent used in this model. This dose of L-NAME does cause a modest rise in blood pressure in guinea-pigs, but splanchnic blood flow was not significantly altered,[27] as would be expected. Organ blood flow is not compromised in hypertensive states, blood pressure is raised to maintain adequate organ perfusion when vascular tone is altered.

Although substance P and many other enteric neuropeptides influence mucosal function, the authors could not decipher the precise role of substance P in this model. The availability of non-peptide substance P antagonists for chronic models of inflammation will be invaluable. Until then studies primarily describe observations and not causations. Nevertheless, our determination that the changes in substance P content in chronic ileitis were granulocyte independent provides some insight. The possibility that nitric oxide may play a role is intriguing, but clearly further investigation is needed in order to unravel these complex interactions.

### References

1. Miller MJS, Sadowska-Krowicka H, Chotinaruemol S, Kakkis JL, Clark DA. Amelioration of chronic ileitis by nitric oxide synthase inhibition. *J Pharmacol Exp Therap* 1993; **264**: 11–16.
2. Miller MJS, Zhang X-J, Sadowska-Krowicka H, Chotinaruemol S, McIntyre JA, Clark DA, Bustamante SA. Nitric oxide release in response to gut injury. *Scand J Gastroenterol* 1993; **28**: 149–154.
3. Allgayer H, Deschyver K, Stenson WF. Treatment with 16,16′-dimethyl prostaglandin $E_2$ before and after induction of colitis with trinitrobenzene sulphonic acid in rats decreases inflammation. *Gastroenterology* 1989; **96**: 1290–1300.
4. Morris GP, Beck PL, Herridge MS, Depew WT, Szewczak MR, Wallace JL. Hapten-induced model of chronic inflammation and ulceration in the rat colon. *Gastroenterology* 1989; **96**: 795–803.
5. Costa M, Cuello AC, Furness JB, Franco R. Distribution of enteric neurons showing immunoreactivity for substance P in the guinea-pig ileum. *Neuroscience* 1980; **5**: 323–331.
6. Miller MJS, Sadowska-Krowicka H, Jeng AY *et al.* Substance P levels in experimental ileitis in the guinea-pig: effects of misoprostol. *Am J Physiol* (in press).
7. Sharkey KA. Substance P and calcitonin gene-related peptide (CGRP) in gastrointestinal inflammation. *Ann New York Acad Sci* 1992; **664**: 425–442.
8. Koch TR, Carney JA, Go VLW. Distribution and quantitation of gut neuropeptides in normal intestine and inflammatory bowel disease. *Dig Dis Sci* 1987; **32**: 369–376.
9. Mantyh PW, Catton MD, Boehmer CG, Welton ML, Passaro EP, Maggio JE, Vigna SR. Receptors for sensory neuropeptides in human inflammatory disease: implications for the effector role of sensory neurons. *Peptides* 1989; **10**: 627–645.
10. Miller MJS, Zhang X-J, Gu X, Clark DA. Acute intestinal injury induced by acetic acid and casein. Prevention by intraluminal misoprostol. *Gastroenterology* 1991; **101**: 22–30.
11. Yamada T, Fujimoto K, Tso P, Fujimoto T, Gaginella TS, Grisham MB. Misoprostol accelerates colonic mucosal repair in acetic acid-induced colitis. *J Pharmacol Exp Therap* 1992; **260**: 313–318.
12. Burch RM, Weitzberg M, Blok N *et al.* *N*-(Fluorenyl-9-methoxycarbonyl) amino acids, a class of anti-inflammatory agents with a different mechanism of action. *Proc Natl Acad Sci USA* 1991; **88**: 355–359.
13. Bator JM, Weitzberg M, Burch RM. *N*-[9H-(2,7-Dimethylfluorenyl-9-methoxy)carbonyl]-L-leucine, NPC 15669, prevents neutrophil adherence to endothelium and inhibits $CD_{11b}/CD_{18}$ upregulation. *Immunopharmacology* 1992; **23**: 139–149.
14. Nathan C. Nitric oxide as a secretory product of mammalian cells. *Faseb J* 1992; **6**: 3051–3064.
15. Kubes P, Suzuki M, Granger DN. Nitric oxide: an endogenous modulator of leukocyte adhesion. *Proc Natl Acad Sci USA* 1991; **88**: 4561–4655.
16. Jeng AY, Wong M, Lovato SJ, Erion MD, Gilligan JP. A radioimmunoassay for measuring α-amidating enzyme activity. *Anal Biochem* 1990; **1 85**: 213–219.
17. Konttinen YT, Kemppinen P, Segerberg M, Sorsa T, Saari T, Scari H, Hukkanen M. Reactive oxygen species induced structural alterations of substance P. *Mediators of Inflammation* 1992; **1**: 355–360.
18. Barson DD, Brokaw JJ, Sekizawa K, McDonald DM, Nadel JA. Neutral endopeptidase and neurogenic inflammation in rats with respiratory infections. *J Appl Physiol* 1989; **66**: 2653–2658.
19. Zimmerman BJ, Anderson DC, Granger DN. Neuropeptidase promote neutrophil adherence to endothelial cell monolayers. *Am J Physiol* 1992; **263**: G678–G682.
20. Stead RH, Bienenstock J, Stanisz AM. Neuropeptide regulation of mucosal immunity. *Immunol Rev* 1987; **100**: 333–359.
21. Grisham MB, Ware K, Gilleland Jr HC, Gilleland LB, Abell CL, Yamada T. Neutrophil-mediated nitrosamine formation: role of nitric oxide in rats. *Gastroenterology* 1992; **103**: 1260–1266.
22. Kubes P. Nitric oxide modulates epithelial permeability in the feline small intestine. *Am J Physiol* 199; **262**: G1138–G1142.
23. Nichols K, Krantis A, Staines W. Histochemical localization of nitric oxide-synthesizing neurons and vascular sites in the guinea-pig intestine. *Neuroscience* 1992; **51**: 791–799.
24. Boughton-Smith NK, Evans SM, Whittle BJR, Moncada S. Induction of colonic nitric oxide synthase in a rat model of colitis (Abstract). *Gastroenterology* 1992; **102**: A598.
25. Salter M, Knowles RG, Moncada S. Widespread tissue distribution, species distribution and changes in activity of $Ca^{2+}$-dependent and $Ca^{2+}$-independent nitric oxide synthases. *FEBS Lett* 1991; **291**: 145–149.
26. Ischiropoulos H, Zhu L, Beckman JS. Peroxynitrite formation from macrophage-derived nitric oxide. *Arch Biochem Biophys* 1992; **298**: 446–451.
27. Miller MJS, Munshi UK, Sadowska-Krowicka H, Kakkis JL, Zhang X-J, Eloby-Childress S, Clark DA. Inhibition of calcium-dependent nitric oxide synthase causes ileitis and leukocytosis in guinea-pigs. *Dig Dis Sci* (in press).

ACKNOWLEDGEMENT. This work was supported by a grant from the Crohn's and Colitis Foundation of America, Inc.

**Received 30 March 1993; accepted in revised form 10 May 1993**
